# Cryo-EM structures of a prokaryotic heme transporter CydDC

**DOI:** 10.1093/procel/pwad022

**Published:** 2023-05-05

**Authors:** Chen Zhu, Yanfeng Shi, Jing Yu, Wenhao Zhao, Lingqiao Li, Jingxi Liang, Xiaolin Yang, Bing Zhang, Yao Zhao, Yan Gao, Xiaobo Chen, Xiuna Yang, Lu Zhang, Luke W Guddat, Lei Liu, Haitao Yang, Zihe Rao, Jun Li

**Affiliations:** Shanghai Institute for Advanced Immunochemical Studies and School of Life Science and Technology, ShanghaiTech University, Shanghai 201210, China; National Clinical Research Center for Infectious Disease, Shenzhen Third People’s Hospital, Shenzhen 518112, China; Shanghai Institute for Advanced Immunochemical Studies and School of Life Science and Technology, ShanghaiTech University, Shanghai 201210, China; Shanghai Institute for Advanced Immunochemical Studies and School of Life Science and Technology, ShanghaiTech University, Shanghai 201210, China; Shanghai Institute for Advanced Immunochemical Studies and School of Life Science and Technology, ShanghaiTech University, Shanghai 201210, China; Shanghai Institute for Advanced Immunochemical Studies and School of Life Science and Technology, ShanghaiTech University, Shanghai 201210, China; State Key Laboratory of Medicinal Chemical Biology, Nankai University, Tianjin 300353, China; Shanghai Institute for Advanced Immunochemical Studies and School of Life Science and Technology, ShanghaiTech University, Shanghai 201210, China; National Clinical Research Center for Infectious Disease, Shenzhen Third People’s Hospital, Shenzhen 518112, China; Shanghai Institute for Advanced Immunochemical Studies and School of Life Science and Technology, ShanghaiTech University, Shanghai 201210, China; Shanghai Institute for Advanced Immunochemical Studies and School of Life Science and Technology, ShanghaiTech University, Shanghai 201210, China; National Clinical Research Center for Infectious Disease, Shenzhen Third People’s Hospital, Shenzhen 518112, China; Shanghai Institute for Advanced Immunochemical Studies and School of Life Science and Technology, ShanghaiTech University, Shanghai 201210, China; Shanghai Institute for Advanced Immunochemical Studies and School of Life Science and Technology, ShanghaiTech University, Shanghai 201210, China; Shanghai Institute for Advanced Immunochemical Studies and School of Life Science and Technology, ShanghaiTech University, Shanghai 201210, China; Shanghai Institute for Advanced Immunochemical Studies and School of Life Science and Technology, ShanghaiTech University, Shanghai 201210, China; School of Chemistry and Molecular Biosciences, The University of Queensland, Brisbane, QLD 4072, Australia; National Clinical Research Center for Infectious Disease, Shenzhen Third People’s Hospital, Shenzhen 518112, China; Shanghai Institute for Advanced Immunochemical Studies and School of Life Science and Technology, ShanghaiTech University, Shanghai 201210, China; Shanghai Institute for Advanced Immunochemical Studies and School of Life Science and Technology, ShanghaiTech University, Shanghai 201210, China; National Clinical Research Center for Infectious Disease, Shenzhen Third People’s Hospital, Shenzhen 518112, China; Laboratory of Structural Biology, Tsinghua University, Beijing 100084, China; State Key Laboratory of Medicinal Chemical Biology, Nankai University, Tianjin 300353, China; Innovative Center for Pathogen Research, Guangzhou Laboratory, Guangzhou 510005, China; Shanghai Institute for Advanced Immunochemical Studies and School of Life Science and Technology, ShanghaiTech University, Shanghai 201210, China; National Clinical Research Center for Infectious Disease, Shenzhen Third People’s Hospital, Shenzhen 518112, China

## Dear Editor,

Heme is an essential cofactor required across all kingdoms of life utilized in numerous biological processes, including cellular respiration. CydDC is a prokaryotic ATP-binding cassette (ABC) transporter required for heme assembling in respiratory cytochrome *bd* oxidase (Georgiou et al., 1987; Poole et al., 1989), a promising target for drug discovery (Borisov et al., 2011). CydDC is thought to play a role in maintaining an optimum periplasmic redox poise that is required for the incorporation of heme cofactors (Yamashita et al., 2014). It also suggested that CydDC is involved in heme processing by mediating glutathione/cysteine translocation (Cook and Poole, 2000; Cook et al., 2002), although a role in heme translocation seems unlikely (Yamashita et al., 2014). To date, CydDC has been shown to be important for disulfide bond formation, motility, respiration, and tolerance to nitric oxide and antibiotics (Poole et al., 2019).

**Figure 1.**
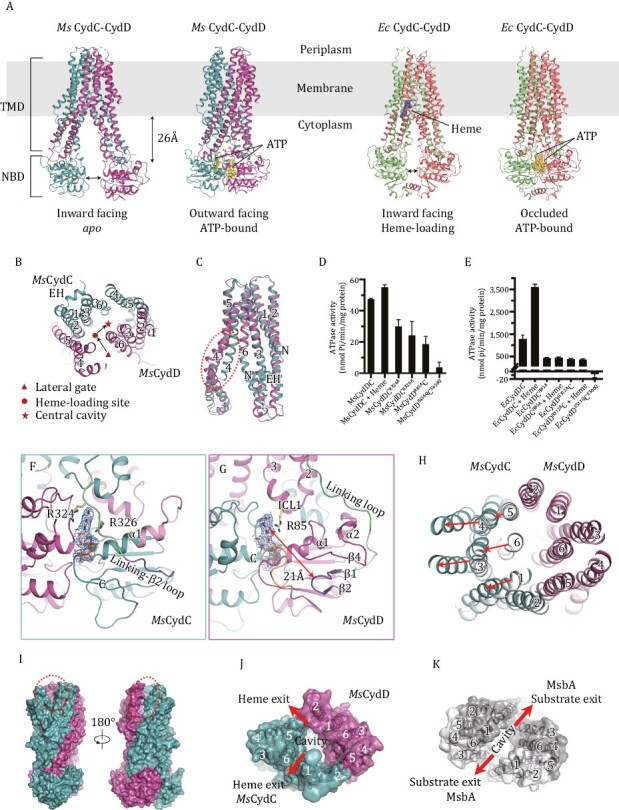
**Overall structures of CydDC, the non-canonical NBDs of *Ms*CydDC and the unique outward-facing conformation.** (A) Overall view of the Cryo-EM structures of the *Msm* CydDC complex in *apo* and ATP-bound states and *Escherichia coli* CydDC complex in heme-loading and ATP-bound states. ATPs and heme are shown as yellow and blue spheres. TMD, transmembrane domain; NBD, nucleotide-binding domain. (B) Clipped view of the TM helices of *apo Ms*CydDC viewed from periplasm. The arrows indicate the path of heme translocation. EH, elbow helix. (C) Superposition of TMDs between *Ms*CydC and *Ms*CydD in the *apo* state. The significant difference is the extent of bending in TM4, indicated by a red dotted circle; N, the N-terminal end. (D and E) The ATPase activity of *Ms*CydDC (D) and *Ec*CydDC (E). Data are mean values with SDs, calculated from three independent experiments. (F and G) The closed view of the non-canonical NBDs of *Ms*CydC (F) and *Ms*CydD (G). The local densities for ATP and Mg^2+^ ion (threshold 0.5) are shown as blue mesh. C indicates the C-terminal end of the polypeptide. Arg85, Arg324 and Arg326 are shown as stick models. (H) The shifts of the TM helices comparing the IF and OF states. The TM helices are viewed from the periplasm. (I) A surface representation of *Ms*CydDC in the outward-facing state. The gaps between TM1^C^ and TM6^C^ and between TM5^C^ and TM2^D^ are marked with a red dotted circle. (J and K) The top view of TM helices in the OF state of *Ms*CydDC (J) and MsbA (PDB code: 3B60) (K). Exiting gaps are indicated.

To further understand the functional role of CydDC in respiratory complex assembly and other physiological processes, we have purified and characterized the CydDC complexes from *Mycobacterium smegmatis* (*Msm*) and *Escherichia coli* (*E*. *coli*) (Fig. S1). The existence of endogenous heme *b* was identified in *Ec*CydDC (Fig. S1C). Both *Ms*CydDC and *Ec*CydDC complexes showed ATPase activities (Fig. 1D and 1E) and among potential substrates, adding additional heme led to a further enhancement of activity in *Ec*CydDC (Figs. 1E, S1D and S1E).

**Figure 2.**
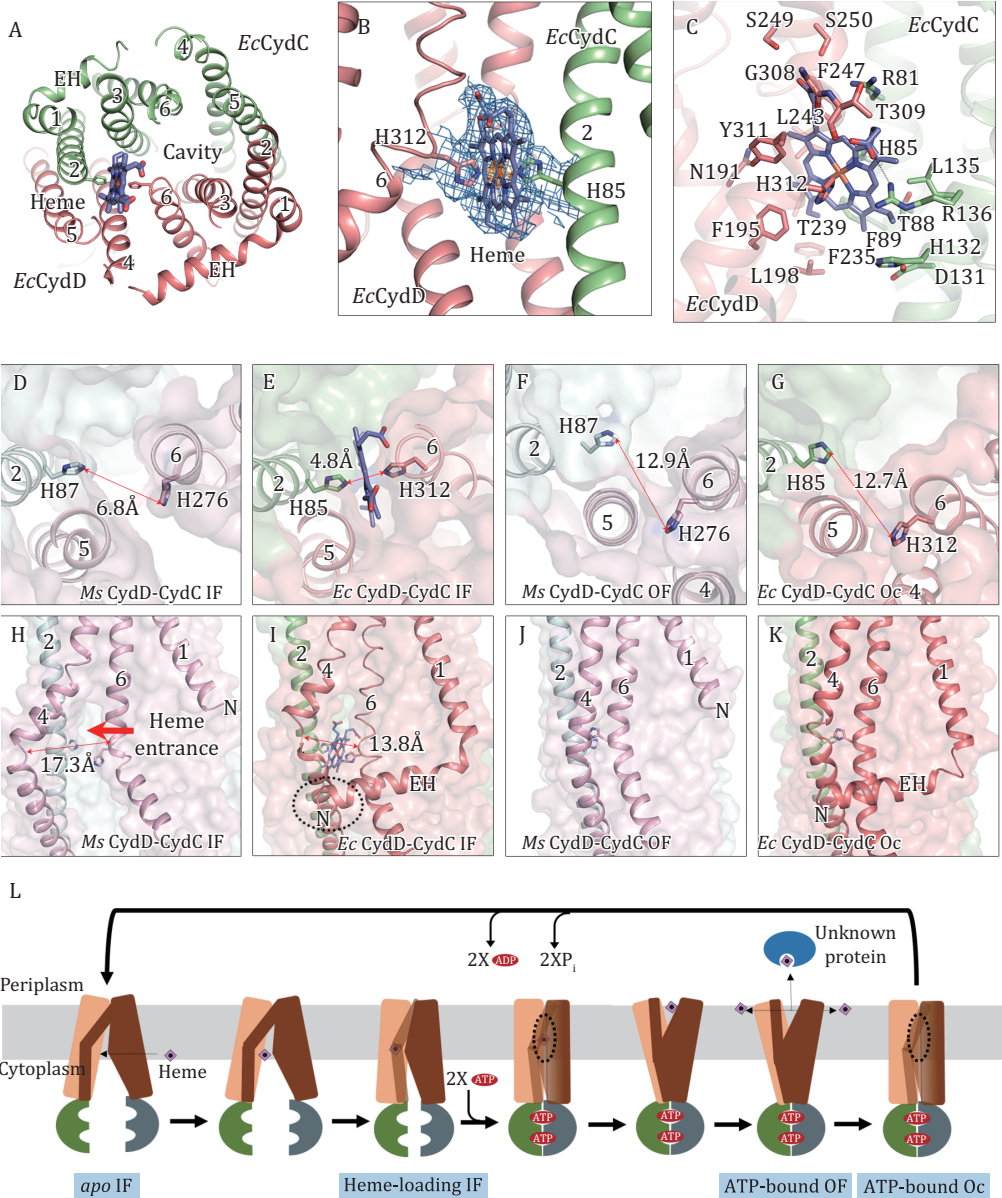
**The heme-loading site, the local conformational changes and proposed mechanism of heme transport by CydDC.** (A) The heme-loading site viewed from periplasm. The heme is shown as stick models. (B) A closed view of heme-load site. Heme is coordinated between axial residues, His85^D^ and His312^C^. The local densities for heme and axial His residues (threshold 0.3) are shown as blue mesh. The local densities for the iron atom in heme (threshold 1.8) is shown as orange mesh. (C) The structure of heme-loading site. Heme and surrounding residues are shown as stick models. Polar interactions are indicated by black dashed lines. (D–G) The distance between two axial His residues in the *apo* IF state of *Ms*CydDC (D), in the heme-loading IF state of *Ec*CydDC (E), in the ATP-bound OF state of *Ms*CydDC (F) and in the ATP-bound Oc state of *Ec*CydDC (G). (H–K) The conformational changes of the lateral gate. (H) The lateral gate between TM4^D^ and TM6^D^ is open for allowing the heme to enter in the *apo* IF state of *Ms*CydDC. (I) TM4^D^ and TM6^D^ are close to each other leaving a small hole that heme is difficult to pass through. The extended EH interacts with TM4^D^. (J) TM4^D^ binds tightly with TM6^D^ in the ATP-bound OF state of *Ms*CydDC. (K) TM4^D^ binds tightly with TM6^D^ in the ATP-bound Oc state of *Ec*CydDC. (L) Proposed mechanism of heme transport by CydDC.

We then determined the Cryo-EM structures of CydDC from *Msm* or *E*. *coli* in the *apo*, heme-loading and ATP-bound states (Figs. 1A, S2–5 and Table S1). In our structures, CydC and CydD form a heterodimer with each subunit composed of one transmembrane domain (TMD) and one nucleotide-binding domain (NBD) (Fig. 1A). TM4 and TM5 of one subunit associate with TM1-3 and TM6 of another to form a helix bundle in a “domain swapping” manner (Fig. 1B). The overall fold of CydDC belongs to the type IV family of ABC transporters, commonly known as exporters (Thomas and Tampe, 2020). The TM1 of *Ms*CydD is short and the Elbow Helix (EH) is missing (Fig. 1C) while the EH of *Ec*CydD is longer than that of CydC (Fig. 2A) and other type IV ABC transporters. Superposition of TMDs in *apo Ms*CydDC shows that the conformation of TM4 is highly bent in CydD (Fig. 1C).

Both the NBDs of *Ms*CydDC are non-canonical. Sequence alignments show that there are deletions in both NBD sequences of *Ms*CydDC compared with other homologs (Fig. S6). In the NBD of *Ms*CydC, half of the Linking Loop, the β1 strand, and the A-loop are missing (Fig. S6A). The rest of the Linking Loop then connects directly with β2, forming a new “Linking-β2 Loop” which passes through the front side of NBD and covers the ATP-binding site (Fig. 1F). Since there is no A-loop in *Ms*CydC to bind ATP, we observed a rescue strategy. Arg324 and Arg326 in the Linking-β2 Loop play an important role in stabilizing ATP by sandwiching its adenosine ring (Fig. 1F). In the NBD of *Ms*CydD, the β1–β2 hairpin becomes really short by deleting a fragment containing the A-loop (Fig. S6B), so that the distance between the adenosine group of ATP and the tip of hairpin (Ala315) is 21 Å (Fig. 1G). To rescue the function of NBD, the adenosine ring of ATP is stabilized by the sidechain of Arg85 from ICL1 located between TM2 and TM3 (Fig. 1G). To confirm the above observations, we performed a mutation analysis and our results showed that a single mutation of any of these Arg residues significantly affected the ATPase activity of *Ms*CydDC (Fig. 1D). This means that these Arg residues are indeed crucial for ATP-binding and they can rescue the function of NBDs in *Ms*CydDC.

At the periplasmic side of TMDs, there are significant shifts for the TM helices in the outward-facing state of *Ms*CydDC. The TM3–TM4 pair, TM5–TM6 pair and TM1 of *Ms*CydC all move away from the center of the helix bundle (Fig. 1H). On the contrary, the arrangement of the TM helices in *Ms*CydD has not changed (Fig. 1H). Thus, the central cavity opens towards the periplasm and gaps are created between TM1^C^ and TM6^C^, and between TM5^C^ and TM2^D^ (Fig. 1I and 1J). These are likely to be the heme exiting paths to the outer leaflet of the membrane. It has also been reported in other outward-open structures such as MsbA (Ward et al., 2007; Lyu et al., 2022) that two large gaps for substrate exiting are formed between TM1 and TM6 of each subunit (Fig. 1K). However, the outward-open manner in CydDC is different to the previously reported outward-open structures even though they have similar folds. Importantly, this alternative substrate exiting channel has never been observed before in the type IV family ABC transporters.

In the Cryo-EM map of heme-loading *Ec*CydDC, we observed a clear density of heme molecule enclosed by TM4–6 of *Ec*CydD and TM2–3 of *Ec*CydC (Fig. 2A and 2B). However, the location of heme is not in the middle of TM region or in the central cavity. This is different from other known ABC transporters where the substrates always bind in the center of the cavity. Crucially, two axial His residues, His85 in TM2^C^ and His312 at the bending point of TM6^D^, coordinate axially to the central iron atom of the heme from opposite sides (Fig. 2B and 2C). Such a binding pattern, is commonly observed in many heme-associated enzymes (Gong et al., 2018; Gong et al., 2020; Wang et al., 2021; Zhou et al., 2021), but never seen in other heme transporters. Mutation of either axial His residues abolished the enhancement of ATPase activity (Fig. 1E). The tetrapyrrole group in heme is further immobilized by many surrounding residues (Fig. 2C).

We then compared the conformational differences around the heme-loading site in different structural states. In the heme-loading structure, the two axial His residues are 4.8 Å away from each other (Fig. 2E), which is perfect to clamp the iron ion. However, in the *apo* state structure, the corresponding His residues are 6.8 Å apart (Fig. 2D) and they can no longer coordinate to the iron center. In the ATP-bound structures, the two axial His residues are 12.9 Å far away from each other in *Ms*CydDC (or 12.7 Å in *Ec*CydDC) (Fig. 2F and 2G). The axial His276 in *Ms*CydD (or His312 in *Ec*CydD) becomes buried among TM4–6 helices (Fig. 2F and 2G). We can infer that when ATP binds, the coordinated heme is released into the central cavity and moves out when its periplasmic side opens.

Next, we tried to find the entrance for the heme binding site. In the *apo* structure of *Ms*CydDC, there is a “lateral gate” formed between TM4^D^ and TM6^D^ near the cytoplasmic side. The distance between the bending points of the two helices is 17.3 Å (Fig. 2H). Thus, it is wide enough to let heme pass through. In the heme-loading structure of *Ec*CydDC, the corresponding distance is shortened to 13.8 Å (Fig. 2I). There is still a hole left, leading to the heme binding site (Fig. 2I). However, it is not large enough to allow the heme to pass through. In the ATP-bound structures of CydDC, such a gap disappears because TM4^D^ and TM6^D^ tightly interact with each other (Fig. 2J and 2K). Interestingly, the long EH of *Ec*CydD protrudes and interacts with TM4^D^ in the heme-loading structure (Fig. 2I). However, *Ms*CydD lacks the whole EH and the gate is largely open in the *apo* structure (Fig. 2H). We therefore infer that EH in CydD may affect the opening of the entrance between TM4^D^ and TM6^D^ and stabilize the heme-loading conformation.

Based on the above analysis, we are able to propose a mechanism of heme transport by CydDC (Fig. 2L): (i) The lipophilic heme synthesized in the cytoplasm is incorporated into the inner leaflet of the membrane and freely solubilized in the hydrophobic lipid environment. (ii) It enters the inward-open *apo* state of CydDC through the wide-open lateral gate between TM4^D^ and TM6^D^. The heme molecule then reaches the loading position. (iii) In the heme-loading site, the two axial His residues get close to each other and clamp the heme. The lateral gate is nearly closed leaving only a hole which will not allow heme to pass through. (iv) ATP molecules bind to the NBDs of both subunits and induce their dimerization. This further triggers a large rearrangement of the TM helices. The lateral gate is totally closed and the central cavity is occluded. The two axial His residues move apart to release heme into the cavity. (v) At the periplasmic side, the interactions between TM helices are not stable and two exiting gaps are formed between TM1^C^ and TM6^C^ and between TM5^C^ and TM2^D^. The cavity opens towards the periplasm and heme can exit through these gaps and be released into the outer leaflet of the membrane, or captured by an unknown protein. Finally, heme is processed and incorporated into cytochrome *bd* as heme *b* or heme *d* (Fig. S7A). (vi) After ATP hydrolysis, the P_i_ and ADP are released from NBDs, the overall conformation of CydDC is returned to the inward-open *apo* state, ready for the next-round of heme transport.

Note that similar mechanism has also been reported by the other group based on a series of *Ec*CydDC models (Wu et al., 2022). Both our and their studies could compensate with each other for heme transport. Besides, we have more findings from the *Ms*CydDC structures such as rescue mechanisms of the non-canonical NBDs and new exiting gaps in the unique outward-open conformation of TMDs.

In conclusion, we determined the Cryo-EM structures of CydDC from either *Msm* or *E*. *coli*, in the *apo* and heme-loading and ATP-bound states. These structures show how the heme is transported across the membrane as a result of conformational changes to the lateral gate, a pair of axial coordinating residues, and its unique exiting gaps, which is critical for the initial stage of respiratory complex assembly. Additionally, the structures identify rescue mechanisms for structural defects in the non-canonical NBDs of *Ms*CydDC, suggesting its functional importance and robustness. Since cytochrome *bd* is a target for the development of antimicrobial drugs, CydDC responsible for its assembly could also be a potential therapeutic target. Thus, our structures provide a framework for the development of new antimicrobial compounds.

## Supplementary Material

pwad022_suppl_Supplementary_Materials

## Footnotes

We thank the staff from the Bio-EM Facility of ShanghaiTech University, for assistance during cryo-EM data collection. We are thankful to the analytical chemistry platform of Shanghai Institute for Advanced Immunochemical studies (SIAIS) and the staff Dr. Jiakang Chen and Dr. Xiuxia Gao for assistance in mass spectrometry analysis. We would like to thank Tianyu Hu, Zhiqi Xiong for the technical help for cryo-EM data processing. This work was supported by grants from National Key R&D Program of China (Grant No. 2021YFA1300900 to J.L.; 2022YFC2302900 to H.Y.), the Lingang Laboratory (Grant No. LG202101-01-08), Shanghai Municipal Commission of Science and Technology (Grant No. 22ZR1441600 to J.L.; ZD2021CY001 to H.Y.) and the Shanghai Frontiers Science Center for Biomacromolecules and Precision Medicine, ShanghaiTech University.

Z.R., J.L. and H.Y. initiated and supervised the project. J.L. designed experiments. C.Z., Y.S., W.Z. and LQ.L. purified the proteins. C.Z. prepared cryo-EM samples and collected the data. C.Z., J.L. solved the cryo-EM structures. C.Z., J.Y. and JX.L. performed biochemical experiments. J.L., C.Z., Y.G., L.W.G., X.C., B.Z., Y.Z., L.Z., XL.Y., XN.Y., L.L., H.Y. and Z.R., analyzed and discussed the results. J.L., C.Z., L.W.G., H.Y. and Z.R. prepared and wrote the manuscript with the help of all the authors.

All data needed to evaluate the conclusions are present in the main text or Supplementary Materials. The accession numbers for the 3D cryo-EM density maps of *Ms*CydDC in *apo* state and ATP-bound state, *Ec*CydDC in heme-loading state and ATP-bound state in this paper are EMD-35640, EMD-35641, EMD-35642 and EMD-35643, respectively. Atomic coordinates for structures of *Ms*CydDC in *apo* state and ATP-bound state, *Ec*CydDC in heme-loading state and ATP-bound state have been deposited in the Protein Data Bank with identification codes: 8IPQ, 8IPR, 8IPS and 8IPT. Additional data related to this paper may be requested from the authors.

The authors declare that they have no competing interests.
