# Supplementary material for: Cryo-EM structures of a prokaryotic heme transporter CydDC
Source: Protein Cell. 2023 May 5;14(12):919–23. doi: 10.1093/procel/pwad022 (PMC10691846; doi:10.1093/procel/pwad022)
Supplement: pwad022_suppl_Supplementary_Materials [file pwad022_suppl_supplementary_materials.pdf]

SUPPLEMENTAL INFORMATION

Cryo-EM structures of a prokaryotic heme transporter CydDC

Chen Zhu[1,2], Yanfeng Shi[1], Jing Yu[1], Wenhao Zhao[1], Lingqiao Li[1], Jingxi Liang[4], Xiaolin Yang[1,2], Bing Zhang[1], Yao Zhao[1,2], Yan Gao[1], Xiaobo Chen[1], Xiuna Yang[1], Lu Zhang[1], Luke W. Guddat[6], Lei Liu[2], Haitao Yang[1,*], Zihe Rao[1,2,3,4,5,*], Jun Li[1,2,*]

[1]Shanghai Institute for Advanced Immunochemical Studies and School of Life Science and Technology, ShanghaiTech University, Shanghai 201210, China

[2]National Clinical Research Center for Infectious Disease, Shenzhen Third People's Hospital, Shenzhen, 518112, China.

[3]Laboratory of Structural Biology, Tsinghua University, Beijing 100084, China

[4]State Key Laboratory of Medicinal Chemical Biology, Nankai University, Tianjin 300353, China

[5]Innovative Center for Pathogen Research, Guangzhou Laboratory, Guangzhou 510005, China

[6]School of Chemistry and Molecular Biosciences, The University of Queensland, Brisbane, QLD 4072, Australia

[*]Correspondence: yanght@shanghaitech.edu.cn (H.Y.), raozh@mail.tsinghua.edu.cn (Z.R.), lijun1@shanghaitech.edu.cn (J.L.)

SUPPLEMENTARY DISCUSSION

In this study, we have determined the Cryo-EM structures of CydDC in different states during substrate transport. During data processing (Figs. S2–5), we found two oligomeric states, one corresponding to a CydDC dimer and the other to a tetramer which could not be separated by the gel filtration (Fig. S1A and S1B). The latter is formed by two copies of CydDC dimer wrapped in one detergent micelle, in an "upside down" or "shoulder to shoulder" manner. For *apo* and ATP-bound *Ms*CydDC and ATP-bound *Ec*CydDC, the maps of tetramers were processed to a high quality while the structure of heme-loading *Ec*CydDC was built from the high-quality map of the dimer (Figs. S2–5). In each of three different models of tetramers, the two copies of CydDC dimer are nearly identical by superposition, thus only one copy was used for structural analysis. The "upside down" form of tetramer seems to be an artifact because the NBDs only exist in the cytoplasmic side of the membrane. While the "shoulder to shoulder" form may possibly exist in the physiological condition yet still needs further evidence. Anyway, the formation of different CydDC tetramers implies that the surface of TM region containing TM3^{D}, TM4^{D}, TM5^{D} and TM2^{C} exhibits high tendency to associate with an unknown membrane protein through the highly hydrophobic interactions. It is interesting that the ATP-bound forms of *Ms*CydDC and *Ec*CydDC are in different conformational states: one in an outward facing conformation and the other in an occluded conformation. Since the residue composition of the TM helices are different, we infer that the TM1^{C}-TM6^{C} interactions and TM2^{D}-TM5^{C} interactions at periplasmic side of *Ms*CydDC are weaker than those of *Ec*CydDC. Thus, the TM1^{C}-TM6^{C} gap and TM2^{D}-TM5^{C} gap are formed in *Ms*CydDC rather than *Ec*CydDC after ATP induced conformational changes in the detergent micelle. In this study, we also observed that heme could stimulate the ATPase activity of *Ec*CydDC (Fig. 1E). This could be explained by our structural analysis. The gap between the two NBDs becomes smaller when the heme binds to CydDC (Fig. 1A). As we know, the ATPase activity of an ABC transporter depends on the dimerization of two NBDs. So that the closer the two NBDs, the more frequent the dimerization, then the higher the ATPase activity.

The identification of heme in a loading position inside the TM region, located in the path between the lateral gate and the central cavity (Fig. 1B), suggests that heme acts as a true substrate of CydDC rather than an allosteric regulator or a cofactor. To allow heme to enter and load in the TM region, CydDC adopts unique features such as highly bent TM helices (i.e. TM4^{D} and TM6^{D}) and an extended EH^{D}. The mode of heme binding in CydDC is unique compared to other reported heme transporters such as *Corynebacterium diphtheriae* HrtBA(Nakamura et al. 2022), *Yersinia pestis* HmuUV(Woo et al. 2012) and Human ABCB6(Kim et al. 2022). In CydDC, the two axial His residues coordinate heme in the similar way to that observed in the respiratory enzymes(Gong et al. 2018; Gong et al. 2020;

Wang et al. 2021; Zhou et al. 2021). Whilst in HrtBA, heme is coordinated by the axial Glu residues(Nakamura et al. 2022); in ABCB6 by the Cys residue in GSH(Kim et al. 2022); and in HmuUV, no coordinating residue is required(Woo et al. 2012). Though other residues in the heme-loading site also contribute to heme binding, we propose that heme coordination by two axial His residues is necessary for CydDC to promote the efficiency of heme transport. This conclusion is supported by an increase in ATPase activity upon heme coordination, and disruption of coordination prevents heme capture as well as stimulation of ATPase activity.

It is not known how the *d*-type heme in cytochrome *bd* is synthesized. It has previously been suggested that CydDC may be required for the formation of heme *d* in *bd*-type cytochromes(Georgiou et al. 1987; Goldman and Kranz 2001). In our structure of CydDC, heme *b* binds with the propionic acid group of *c* ring bent which resembles the conformation of heme *d* (Fig. S7). We hypothesize that heme *b* may be oxidized by a radical species free in the membrane to form heme *d* when loading in CydDC during transport. This is based on the model of heme *d* production by hydroperoxidase catalase II from *E. coli(Obinger et al. 1997)*. However, further evidence will be required to confirm this hypothesis.

The fold of the NBDs in ABC-type transporters is very conserved, especially for the Walker A, Walker B, A-loop and Q-loop which are responsible for ATP binding and hydrolysis. A deletion or mutation in the conserved NBD affects or damages its function. We have observed rare deletions in both NBDs of *Ms*CydDC which probably occurred as this bacterium evolved, but they are not observed in other closely related mycobacterial homologs (Fig. S6). Unexpectedly, *Ms*CydDC has lost some key structures for both NBDs including the A-loop which is important for ATP binding and hydrolysis. However, structural rearrangement and the evolved presence of Arg residues outside of NBDs to stabilize ATP is successful for rescuing the function of *Ms*CydDC, though its ATPase activity is largely reduced compared to *Es*CydDC. We believe that similar strategies could be generally adopted during protein evolution. As a consequence, the function of one subunit may be dependent on cooperating with other parts of the complex. The rescue mechanism in *Ms*CydDC also shows the functional importance of CydDC for bacteria and the robustness of this protein complex. On the other hand, our findings of the non-canonical NBDs represent the observation of new folds for an NBD in the ABC transporter family.

Since cytochrome *bd* is a target for the development of antimicrobial drugs, CydDC responsible for its assembly could also be a potential therapeutic target. It has been shown that disruption of *cydC* in *Mtb* accelerated bacterial clearance in isoniazid-treated mice(Dhar and McKinney 2010), thus a combined treatment of anti-tuberculosis drugs with a CydDC inhibitor could be an effective therapeutic strategy. The structures of CydDC in this study will provide a framework for future drug development efforts.

97

MATERIALS AND METHODS

Cloning and expression

The cluster of *cydD-cydC* genes (*MSMEG_3231* and *MSMEG3240*) from *Msm* strain *mc^{2}155* genome were cloned into the engineered pMV261 vector fused with a flag tag attached to the C-terminus of CydC, under the control of an acetamide promoter. The resultant plasmid was introduced into *Msm mc^2 155* competent cells by electroporation. The cells were cultivated in Luria-Bertani broth (LB) liquid media supplemented with 50 μg mL^{-1} kanamycin, 20 μg mL^{-1} carbenicillin and 0.1% (v/v) Tween80. When the cells were grown to an optical density (OD_{600}) of 0.6~0.8 at 37 ℃, overexpression of the recombinant protein was induced by 0.2% (w/v) acetamide at 16 ℃. After four days, cells were harvested by centrifugation and frozen at -80 ℃. All mutants of *Ms*CydDC were expressed using the same protocol as the wild-type protein. The cluster of *cydD-cydC* genes (*ECBD_2708* and *ECBD_2709*) from the *E. coli* BL21 genome were cloned into the pET-22b vector fused with a flag tag attached to the C-terminus of CydC. After the resultant plasmid was transformed into *E. coli* BL21 (DE3) cells, cells were grown in LB medium supplemented with 50 μg mL^{-1} ampicillin at 37 ℃. When the OD_{600} reached 0.8, induction of target expression was with 0.3 mM isopropyl-β-D-thiogalactopyranoside (IPTG). The culture was further incubated at 16 ℃ for 16 h. Cells were harvested by centrifugation and frozen at -80 ℃. All mutants of *Ec*CydDC were expressed using the same protocol as the wild-type protein.

Protein purification

To purify the *Ms*CydDC complex, cell pellets were thawed and resuspended in Buffer A (20 mM Tris-HCl, pH 8.0, 300 mM NaCl, 100 mM KCl, 4 mM $MgCl_2$), and then lysed by passing through a French Press at 1,200 bar. Cell debris was removed by centrifugation at 12,000 rpm for 12 min at 4 ℃. The supernatant was collected and ultra-centrifuged at 150,000 *g* for 1 h at 4 ℃. The membrane fraction was resuspended in Buffer A and incubated for 1.5 h with 1% (w/v) Laurylmaltose neopentylglycol (LMNG; Anatrace) at 4 ℃. The suspension was ultra-centrifuged and the supernatant was applied to anti-FLAG (Sigma) affinity beads and washed with Buffer A supplemented with 0.004% (w/v) LMNG. To exchange LMNG to glyco-diosgenin (GDN; Anatrace), the beads were washed with 40 mL Buffer A supplemented with 0.02% (w/v) GDN. The recombinant protein complex was eluted from the beads with Buffer A supplemented with 0.2 mg mL^{-1} peptide (DYKDDDK) and 0.02% (w/v) GDN. The eluted sample was concentrated and then applied to a size exclusion chromatography column (Superose-6 increase, GE Healthcare) pre-equilibrated with Buffer A complemented with 0.02% (w/v) GDN and 4 mM DTT. The peak fractions containing protein complex were pooled and concentrated. All mutants of *Ms*CydDC were purified following a similar protocol to the wild-type complex. To obtain the samples of *Ec*CydDC complex and

its mutants, N-dodecyl-b-D-maltoside (DDM; Anatrace), instead of LMNG and GDN, was used for protein purification. The rest of the procedure was the same as for *Ms*CydDC.

ATPase assay

All ATPase assays were performed using the ATPase/GTPase Activity Assay Kit (Sigma-Aldrich, catalog number MAK113-1KT) as described previously(Liu et al. 2020). A total of 5 μL of purified *Ms*CydDC (1 μM) or *Ec*CydDC (0.2 μM) was incubated with 15 μL buffer A supplemented with 8 mM ATP at 37 ℃ for 2 min. The reaction was stopped by adding 100 μL of reagent (catalog number MAK113A) and incubated for an additional 30 min at room temperature to generate the colorimetric product. Absorbance at 620 nm was measured at room temperature using a CLARIOstar plate reader (BMG LabTech). ATPase activity was represented as the amount of phosphate (nanomoles) produced by 1 mg of protein per minute. All experiments were performed in triplicate. To test the response of the ATPase activity to potential substrates, GSH, GSSG or Cys was added to the reaction mixture to the final concentration of 0.5 mM. Heme was prepared as describe previously(Owens et al. 2013) and added to the reaction mixture in a 5:1 molar concentration ratio to the protein.

Cryo-EM sample preparation and data acquisition

Purified *Ms*CydDC was concentrated to 4 mg mL^{-1} and 3 μL sample was applied to H_2/O_2 glow-discharged holey carbon grids (Cu300 1.2/1.3, Quantifoil). Excess liquid was removed by blotting for 4 s (blot force −1) using filter paper followed by plunge freezing in liquid ethane using the Vitrobot at 7 ℃ and 100% humidity. The *Ms*CydD^{E456Q}C^{E456Q} was incubated with 10 mM ATP at room temperature for 3 min (*Ec*CydD^{E511Q}C^{E500Q} was incubated with 4 mM ATP at 4 ℃ for 30 min) before preparing the Cryo-EM sample. The *Ms*CydDC sample was imaged in a 300 kV FEI Titan Krios (Thermo Fisher Scientific) electron microscope with a Gatan K3 Summit direct electron detector (Gatan) and a Gatan Quantum energy filter at ×130,000 nominal magnification. Data were collected in super-resolution mode at a pixel size of 0.82 Å (0.96 Å for *Ec*CydDC) with a total dose of 60 $e^-/Å^2$. SerialEM(Mastronarde 2005) was used to automatically acquire data. The details of electron microscopy data collection parameters for each batch of *Ms*CydDC or *Ec*CydDC complexes are provided (Table S1).

EM data analysis

All dose-fractionated image stacks were motion-corrected and dose-weighted using MotionCorr2 software(Zheng et al. 2017). CTF estimation was performed using cryoSPARC(Punjani et al. 2017). Micrographs that exhibited defects in the Thon rings due to excessive drift, ice contamination, or astigmatism were discarded. For the *apo* *Ms*CydDC dataset, 1,306,165 particles are picked and auto-extracted from 3805 micrographs with a box

size of 320 pixels. Then the particles are submitted to reference-free 2D classification to screen out the integrated particles and 246,608 particles were used to create 3D models in *ab initio* reconstruction process. After several rounds of hetero refinement, a best 3D model with 107,090 particles was picked out and submitted to non-uniform refinement to generate the final cryo-EM map with an estimated average resolution of 3.5 Å according to the gold-standard Fourier shell correlation cutoff of 0.143(Grigorieff 2016). Local resolution ranges were also analyzed within cryoSPARC. The datasets for ATP-bound *Ms*CydDC, heme-loading *Ec*CydDC and ATP-bound *Ec*CydDC were processed in the same way.

Model building and refinement.

The atomic model was manually built in Coot(Emsley et al. 2010) based on the cryo-EM map. ATP and heme were fitted into the cryo-EM map according to the additional non-protein density. The model was submitted to Phenix(Adams et al. 2013) to perform real-space refinement and validation with secondary structure and geometry restraints to prevent over-fitting. For the mutant proteins, the model of the wild-type protein was used as reference and fitted to the cryo-EM map using Chimera(Pettersen et al. 2004). The refinement statistics of the final models are shown in Table S1. All figures were made using PyMOL (The PyMOL molecular graphics system, Schrödinger, LLC.)

Mass spectrometry

Pre-chilled acetone was added to protein samples in a ratio of 4:1, then mixed, incubated at -20 ℃ for 4 h, then centrifuged at 12000 rpm at 4 ℃ for 30 min. The resulting supernatant was vacuum drained and solubilized in 0.1% FA and then loaded onto a ACE C 18 20*2.1 5 μM column connected to Shimadzu 30A mass spectrometer. Analysis was performed using the ABScix 4600 software. The elution gradient and mobile phase constitution used for heme separation were as follows: 0-0.5 min, 5% B; 0.5-6 min, 5-95% B; 6-8 min, 95% B (mobile phase A: 0.1% formic acid in water; mobile phase B: 0.1% formic acid in CH_3CN) at a flow rate of 0.6 mL min^{-1}. The mass spectrometer was operated in positive mode. The source voltage, curtain gas, and source temperature were set to 5500 V, 35 psi and 350 ℃, respectively.

References

Adams, P. D., et al. (2013), 'Advances, interactions, and future developments in the CNS, Phenix, and Rosetta structural biology software systems', *Annu Rev Biophys,* 42, 265-87.

Dhar, N. and McKinney, J. D. (2010), 'Mycobacterium tuberculosis persistence mutants identified by screening in isoniazid-treated mice', *Proc Natl Acad Sci U S A,* 107 (27), 12275-80.

Emsley, P., et al. (2010), 'Features and development of Coot', *Acta Crystallogr D Biol Crystallogr,* 66 (Pt 4), 486-501.

Georgiou, C. D., Fang, H., and Gennis, R. B. (1987), 'Identification of the cydC locus required for expression of the functional form of the cytochrome d terminal oxidase complex in Escherichia coli', *J Bacteriol,* 169 (5), 2107-12.

Goldman, B. S. and Kranz, R. G. (2001), 'ABC transporters associated with cytochrome c biogenesis', *Res Microbiol,* 152 (3-4), 323-9.

Gong, H., et al. (2020), 'Cryo-EM structure of trimeric Mycobacterium smegmatis succinate dehydrogenase with a membrane-anchor SdhF', *Nat Commun,* 11 (1), 4245.

Gong, H., et al. (2018), 'An electron transfer path connects subunits of a mycobacterial respiratory supercomplex', *Science,* 362 (6418).

Grigorieff, N. (2016), 'Frealign: An Exploratory Tool for Single-Particle Cryo-EM', *Methods Enzymol,* 579, 191-226.

Kim, S., et al. (2022), 'Structural Insights into Porphyrin Recognition by the Human ATP-Binding Cassette Transporter ABCB6', *Mol Cells,* 45 (8), 575-87.

Liu, F., et al. (2020), 'Structural basis of trehalose recycling by the ABC transporter LpqY-SugABC', *Sci Adv,* 6 (44).

Mastronarde, D. N. (2005), 'Automated electron microscope tomography using robust prediction of specimen movements', *J Struct Biol,* 152 (1), 36-51.

Nakamura, H., et al. (2022), 'Structural basis for heme detoxification by an ATP-binding cassette-type efflux pump in gram-positive pathogenic bacteria', *Proc Natl Acad Sci U S A,* 119 (27), e2123385119.

Obinger, C., et al. (1997), 'Activity, peroxide compound formation, and heme d synthesis in Escherichia coli HPII catalase', *Arch Biochem Biophys,* 342 (1), 58-67.

Owens, C. P., et al. (2013), 'The Mycobacterium tuberculosis secreted protein Rv0203 transfers heme to membrane proteins MmpL3 and MmpL11', *J Biol Chem,* 288 (30), 21714-28.

Pettersen, E. F., et al. (2004), 'UCSF Chimera--a visualization system for exploratory research and analysis', *J Comput Chem,* 25 (13), 1605-12.

Punjani, A., et al. (2017), 'cryoSPARC: algorithms for rapid unsupervised cryo-EM structure

determination', *Nat Methods,* 14 (3), 290-96.
Wang, W., et al. (2021), 'Cryo-EM structure of mycobacterial cytochrome bd reveals two oxygen access channels', *Nat Commun,* 12 (1), 4621.
Woo, J. S., et al. (2012), 'X-ray structure of the Yersinia pestis heme transporter HmuUV', *Nat Struct Mol Biol,* 19 (12), 1310-5.
Zheng, S. Q., et al. (2017), 'MotionCor2: anisotropic correction of beam-induced motion for improved cryo-electron microscopy', *Nat Methods,* 14 (4), 331-32.
Zhou, X., et al. (2021), 'Architecture of the mycobacterial succinate dehydrogenase with a membrane-embedded Rieske FeS cluster', *Proc Natl Acad Sci U S A,* 118 (15).

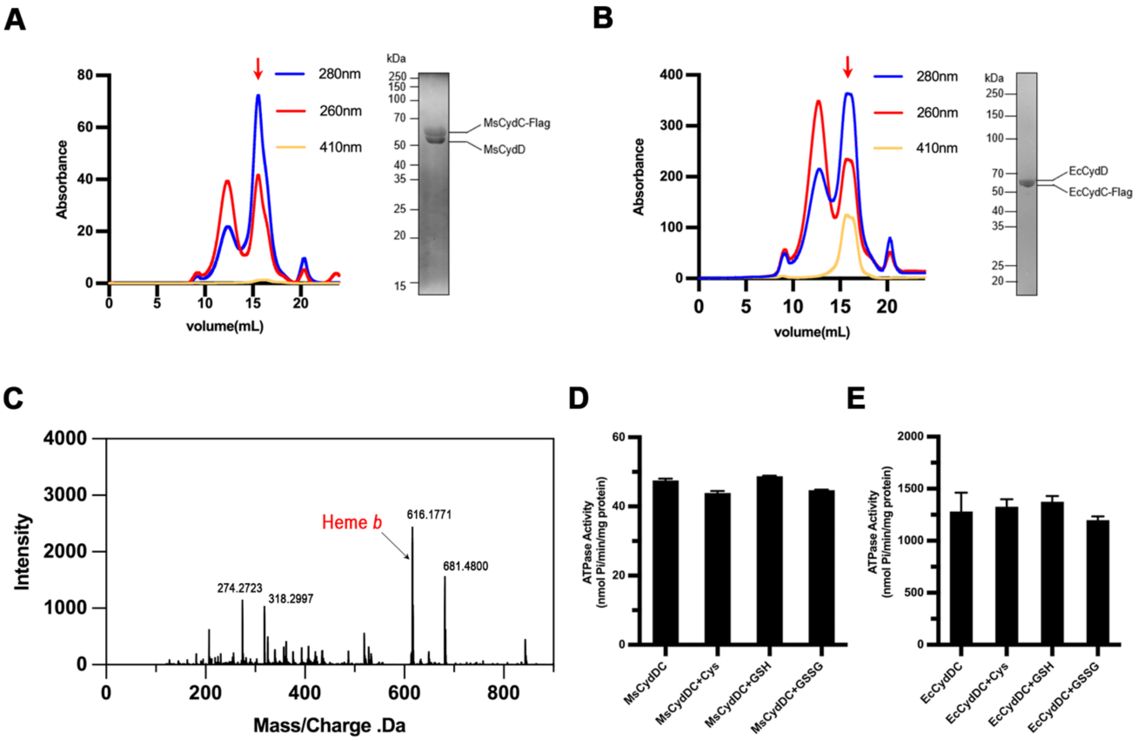

Figure S1 Characterization of CydDC

(A-B) Superose 6 gel filtration column chromatography (GE healthcare) for the *Ms*CydDC (A) and *Ec*CydDC (B) and the SDS-PAGE of the eluted peak indicated by the red arrow. (C) Mass spectrometry analysis identified that heme *b* (MW: 616.5 Da) co-purified in the *Ec*CydDC complex. (D-E) The ATPase activity of *Ms*CydDC (D) and *Ec*CydDC (E) with Cys, GSH and GSSG. Data are presented as mean values with SDs, calculated from three independent experiments.

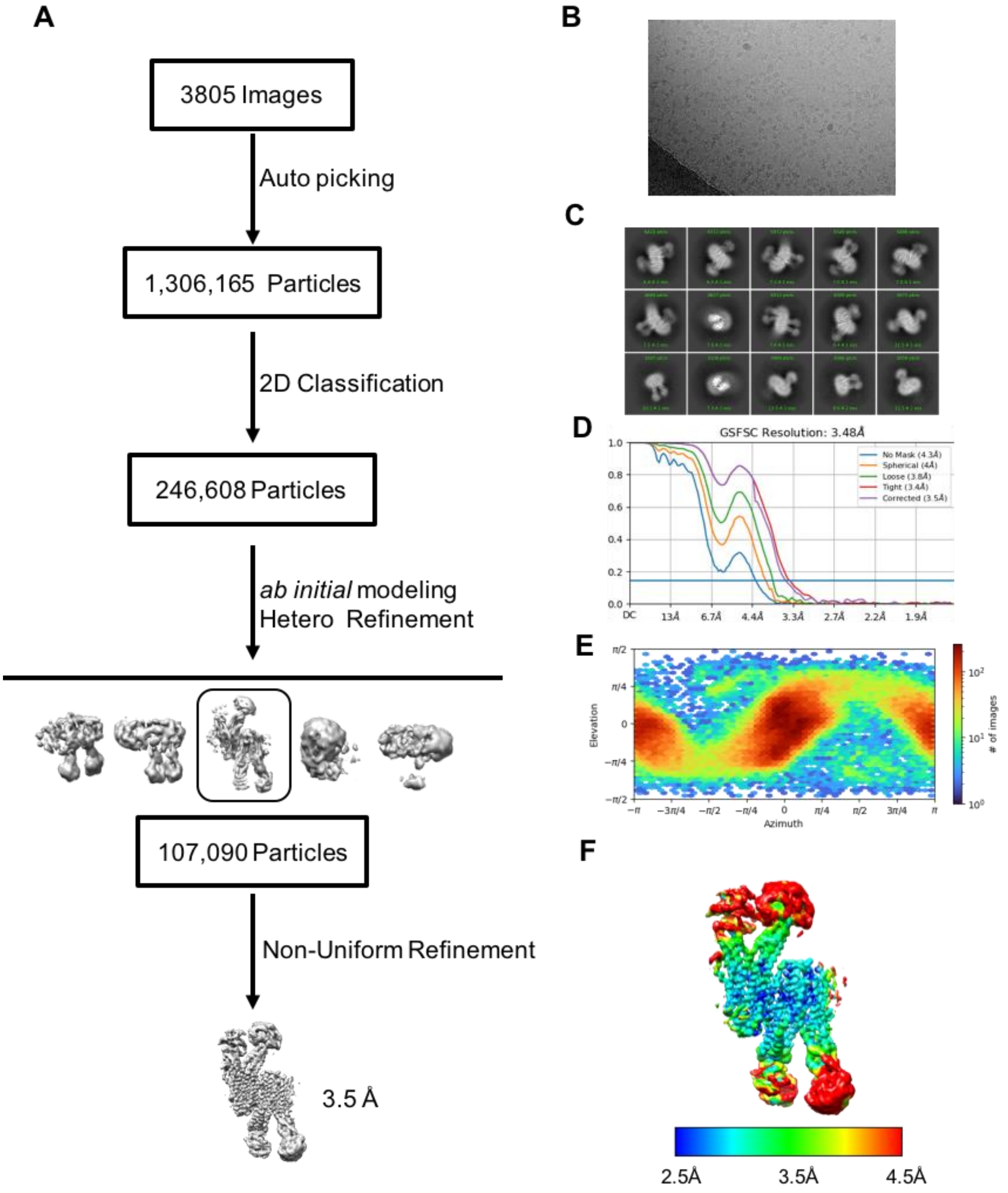

Figure S2 Cryo-EM data processing of the *apo Ms*CydDC

(A) Flow chart for the processing of cryo-EM data. (B) Representative electron micrograph. (C) Selected reference-free 2D class averages. (D) Gold-standard Fourier correlation curves of 3D reconstructions. (E) Posterior precision directional distributions of all particles used in the final 3D reconstruction generated by cryoSPARC. (F) The density map colored according to the local resolution estimate using cryoSPARC.

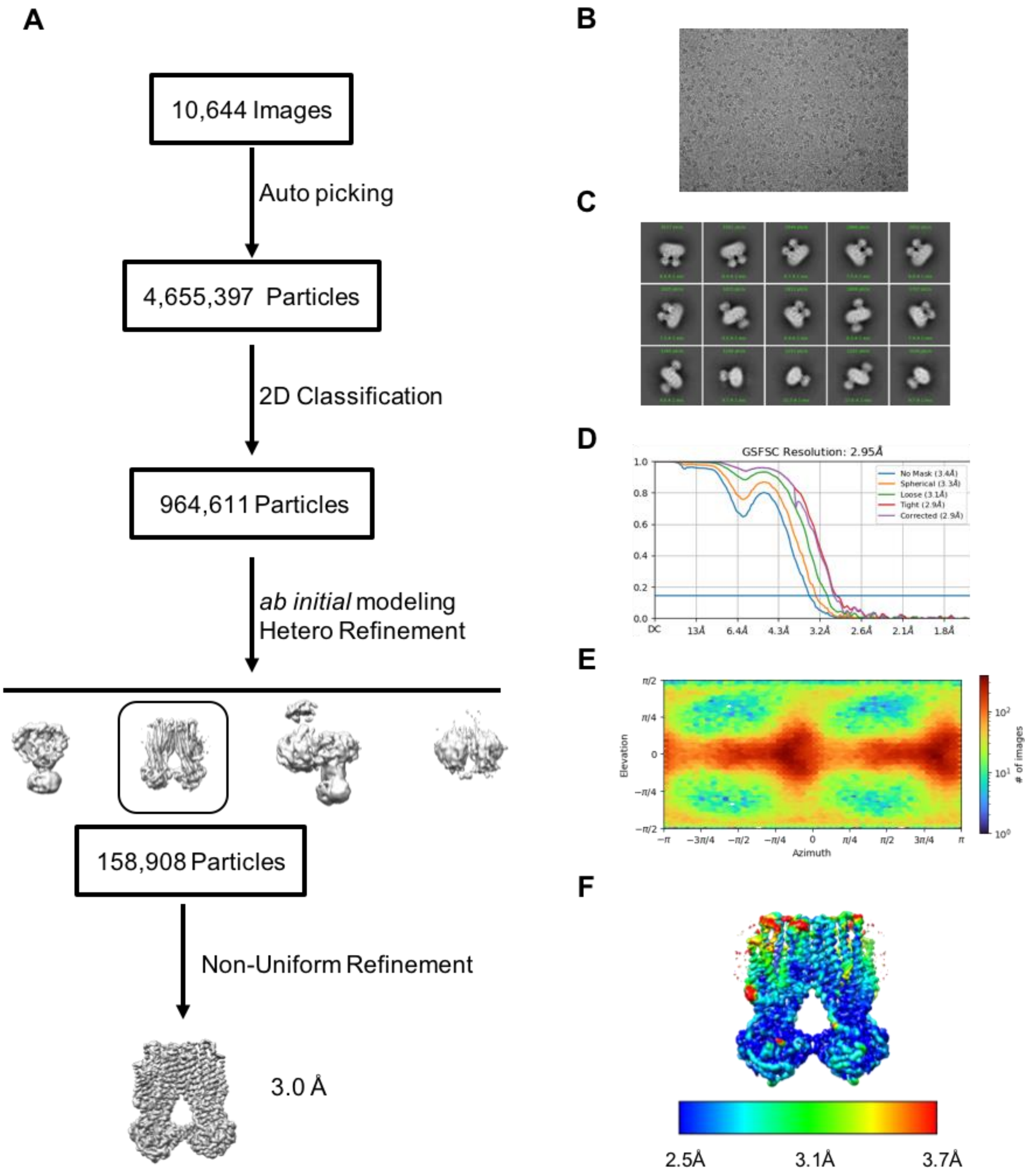

Figure S3 Cryo-EM data processing of the ATP-bound *Ms*CydDC

(A) Flow chart for the processing of cryo-EM data. (B) Representative electron micrograph. (C) Selected reference-free 2D class averages. (D) Gold-standard Fourier correlation curves of the 3D reconstructions. (E) Posterior precision directional distributions of all particles used in the final 3D reconstruction generated by cryoSPARC. (F) The density map colored according to the local resolution estimate using cryoSPARC.

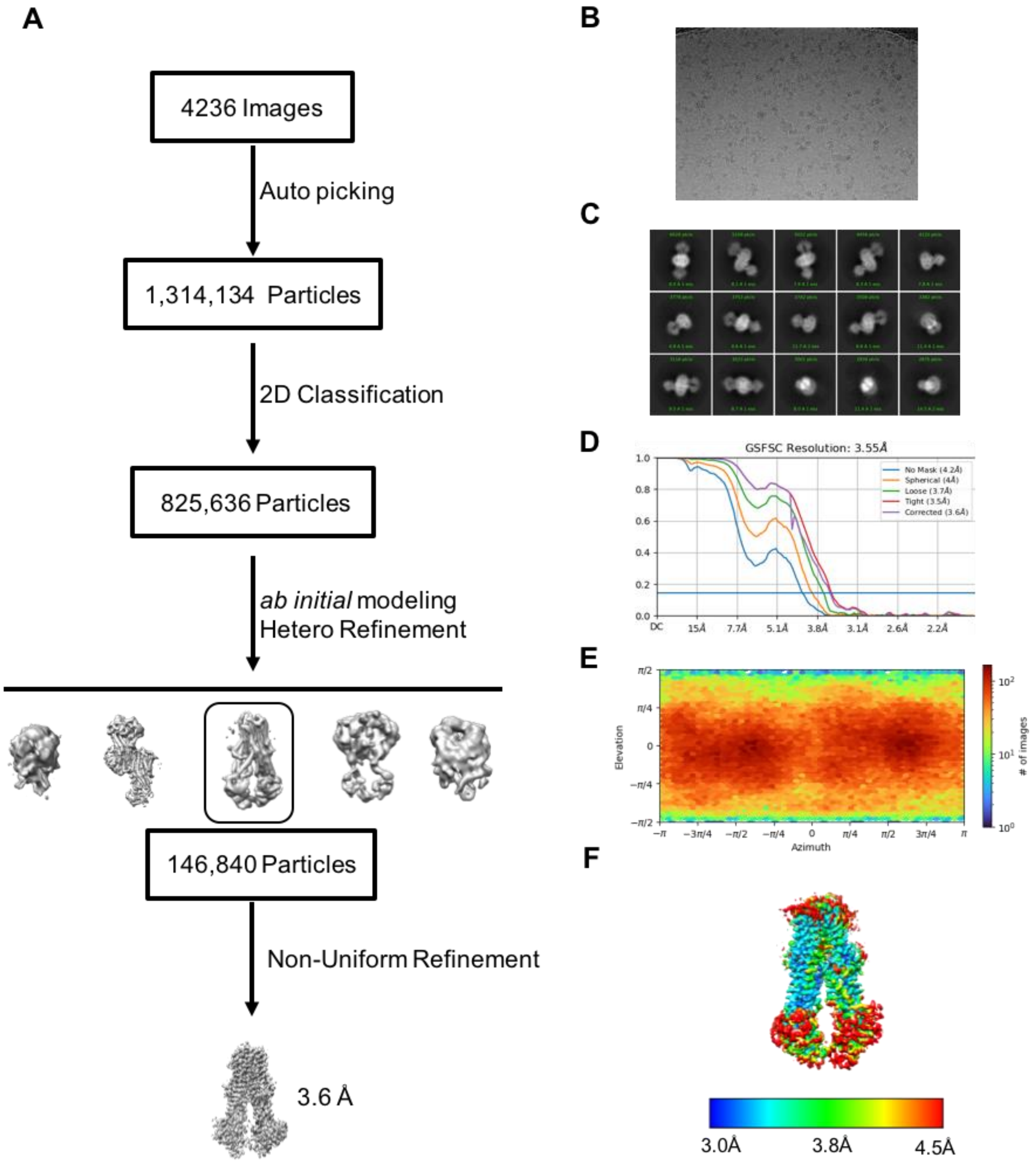

Figure S4 Cryo-EM data processing of the heme-loading *Ec*CydDC
(A) Flow chart for the processing of cryo-EM data. (B) Representative electron micrograph. (C) Selected reference-free 2D class averages. (D) Gold-standard Fourier correlation curves of 3D reconstructions. (E) Posterior precision directional distributions of all particles used in the final 3D reconstruction generated by cryoSPARC. (F) The density map colored according to the local resolution estimate using cryoSPARC.

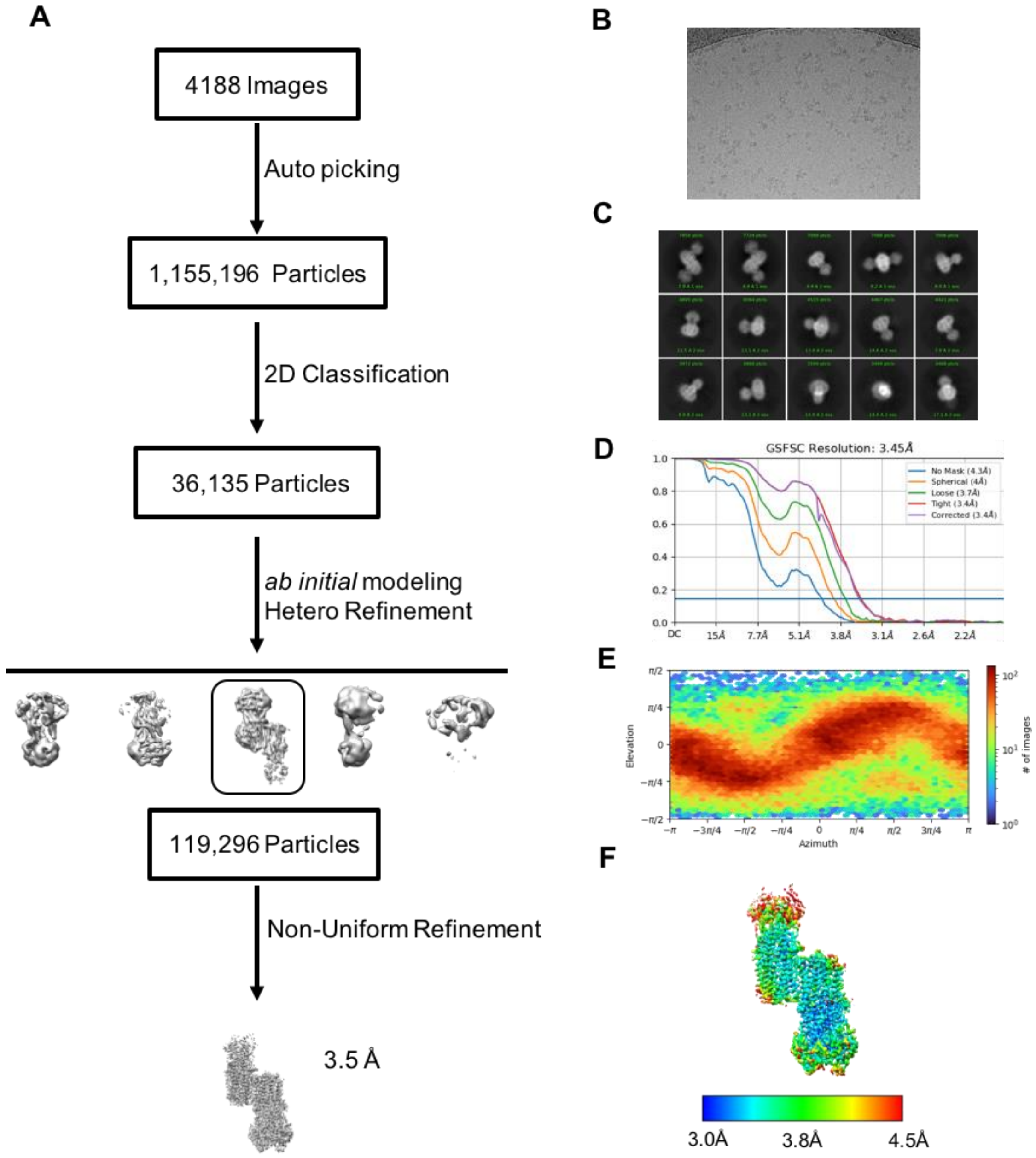

Figure S5 Cryo-EM data processing of the ATP-bound *Ec*CydDC

(A) Flow chart for the processing of cryo-EM data. (B) Representative electron micrograph. (C) Selected reference-free 2D class averages. (D) Gold-standard Fourier correlation curves of 3D reconstructions. (E) Posterior precision directional distributions of all particles used in the final 3D reconstruction generated by cryoSPARC. (F) The density map colored according to the local resolution estimate using cryoSPARC.

A

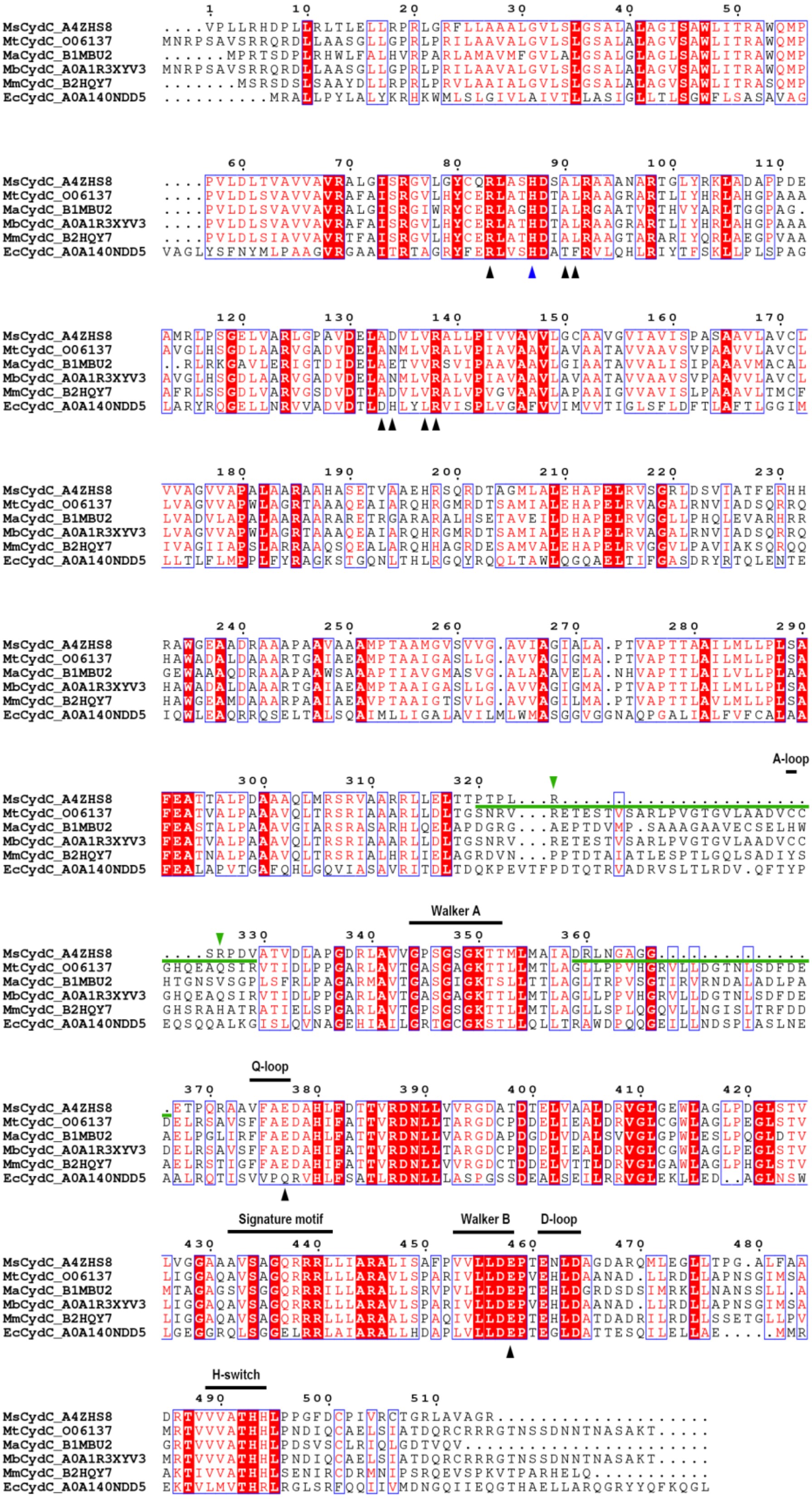
MsCydC_A4ZHS8
MtCydC_O06137
MaCydC_B1MBU2
MbCydC_A0A1R3XYV3
MmCydC_B2HQY7
EcCydC_A0A140NDD5
A-loop
Walker A
Q-loop
Signature motif
Walker B
D-loop
H-switch

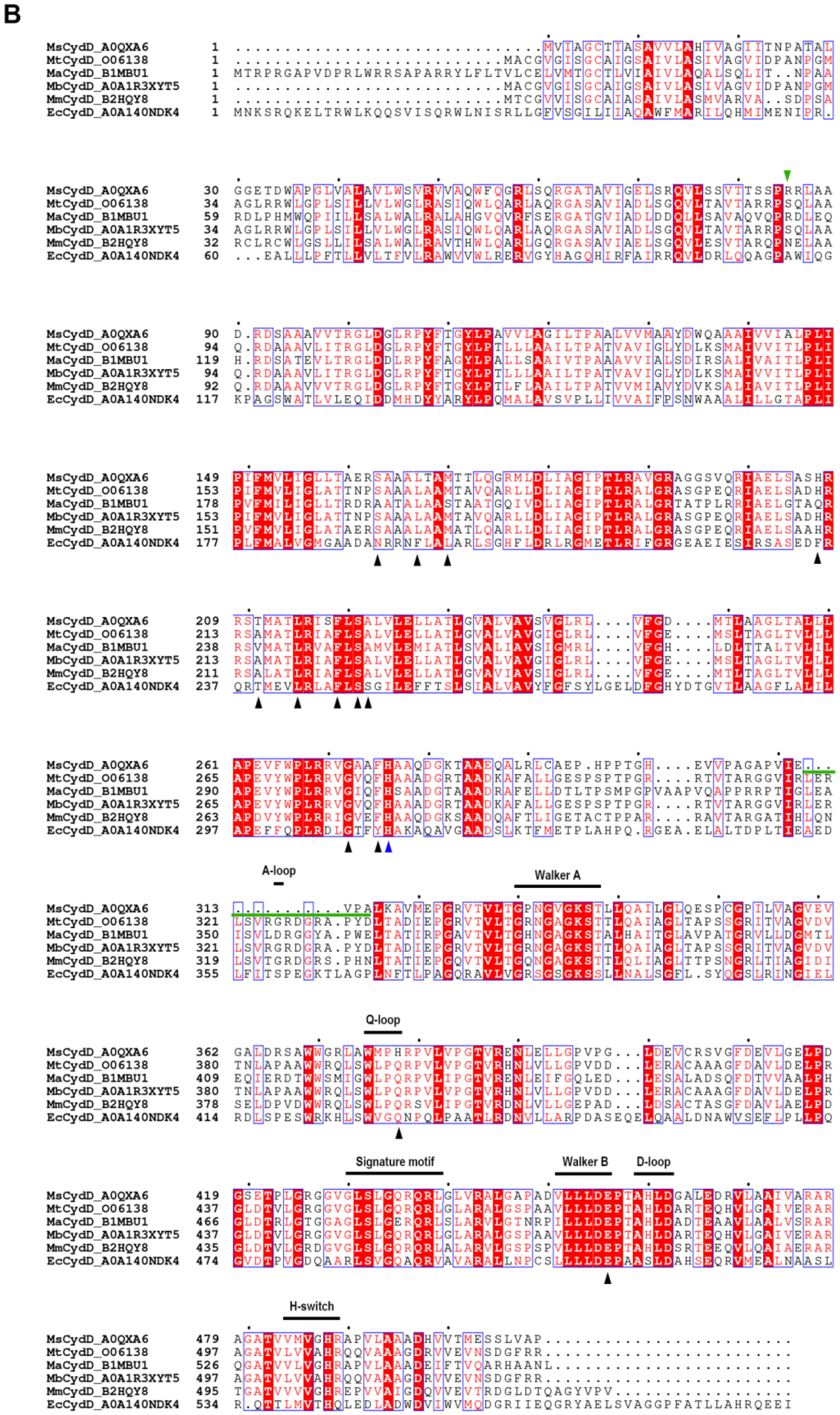

Figure S6 Sequence alignment of CydC and CydD.

(A-B) Sequence alignment of CydC proteins (A) and CydD proteins (B) from *Mycobacterium smegmatis* (Ms), *Mycobacterium tuberculosis* (Mt), *Mycobacterium abscessus* (Ma), *Mycobacterium bovis* (Mb), *Mycobacterium marinum* (Mm) and *Escherichia coli* (Ec). The conserved motifs are marked with bars on the top of the aligned sequences. The residues discussed in the text are highlighted with triangles. The deletions in *Ms*CydC and *Ms*CydD are underlined.

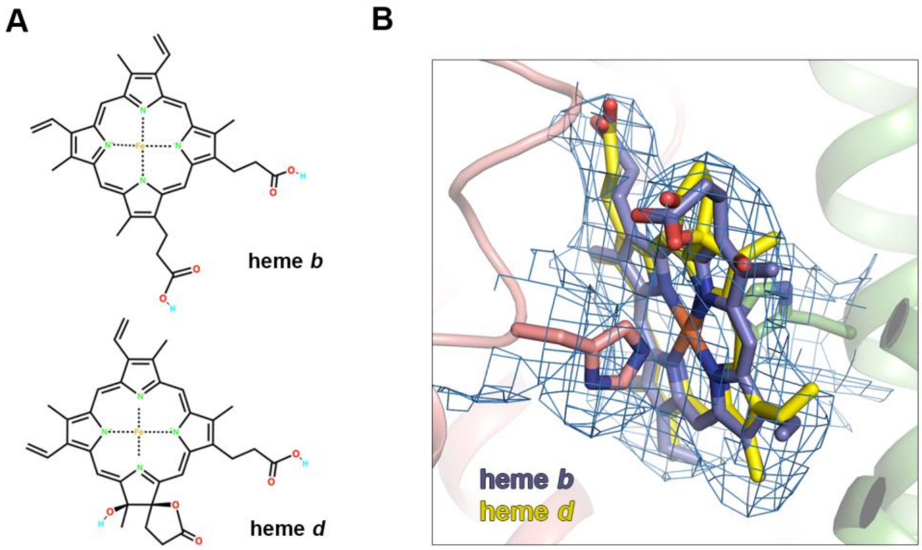

Figure S7 Structures of heme *b* and heme *d*.

(A) The chemical structures of heme *b* and heme *d*. (B) Heme *d* was fitted into the map (threshold 0.3) in agreement with how heme *b* binds in the heme-loading state of *Ec*CydDC.

Table S1 Cryo-EM data collection, refinement and validation statistics.

	Ms*CydDC *apo	***Ms*CydD^{E456Q}C^{E458Q}+ATP**	***Ec*CydDC +Heme**	***Ec*CydD^{E511Q}C^{E500Q}+ATP**
Microscope	FEI Titan Krios	FEI Titan Krios	FEI Titan Krios	FEI Titan Krios
Magnification	165,000×	165,000×	105,000×	105,000×
Voltage (keV)	300	300	300	300
Electron exposure (e^-/Å^2)	60	60	60	60
Defocus range (μm)	-1.2 to -1.8	-1.2 to -1.8	-1.2 to -1.8	-1.2 to -1.8
Pixel size (Å/pixel)	0.832	0.832	0.960	0.960
Number of movies	3,805	10,644	4,236	4,188
Symmetry imposed	C1	C2	C1	C1
Final particle images (no.)	107,090	158,908	146,840	84,448
Map resolution (Å)	3.5	3.0	3.6	3.5
FSC threshold	0.143	0.143	0.143	0.143
**Refinement**				
Model resolution (Å)	3.5	2.9	3.5	3.4
FSC threshold	0.143	0.143	0.143	0.143
Map sharpening *B* factor (Å^2)	-116.7	-118.2	-149.0	-89.0
Model composition				
Non-hydrogen atoms	14,456	14,894	9,072	18,178
Protein residues	1,996	2,038	1,161	2,322
Ligands	0	8	1	4
B factors (Å^2)				
Protein	148.51	57.74	41.70	69.02
Ligand	-	31.94	38.56	80.23
R.m.s. deviations				
Bond lengths (Å)	0.004	0.003	0.005	0.004
Bond angles (°)	0.774	0.667	0.703	1.015
Validation				
MolProbity score	2.18	1.97	1.91	2.01
Clash score	16.75	11.28	8.07	9.82
Poor rotamers (%)	0.61	0.13	0.21	0.32
Ramachandran plot				
Favored (%)	92.73	93.99	92.39	91.88
Allowed (%)	7.17	5.86	7.61	7.95
Outliers (%)	0.1	0.15	0.00	0.17

311
